# Diagnostic models for impending death in terminally ill cancer patients: A multicenter cohort study

**DOI:** 10.1002/cam4.4314

**Published:** 2021-09-29

**Authors:** Masanori Mori, Takuhiro Yamaguchi, Isseki Maeda, Yutaka Hatano, Takashi Yamaguchi, Kengo Imai, Ayako Kikuchi, Yosuke Matsuda, Kozue Suzuki, Satoru Tsuneto, David Hui, Tatsuya Morita, EASED collaborators

**Affiliations:** ^1^ Seirei Mikatahara Hospital Hamamatsu Japan; ^2^ Tohoku University Graduate School of Medicine Sendai Japan; ^3^ Senri Chuo Hospital Toyonaka Japan; ^4^ Daini Kyoritsu Hospital Kawanishi Japan; ^5^ Konan Medical Center Kobe Japan; ^6^ Mitsubishi Kyoto Hospital Kyoto Japan; ^7^ St. Luke's International Hospital Tokyo Japan; ^8^ Tokyo Metropolitan Cancer and Infectious Disease Center Komagome Hospital Bunkyo‐ku Japan; ^9^ Kyoto University Graduate School of Medicine Kyoto Japan; ^10^ MD Anderson Cancer Center Houston Texas USA

**Keywords:** advanced cancer, diagnostic models, impending death, recursive partitioning analysis

## Abstract

**Background:**

Accurately predicting impending death is essential for clinicians to clarify goals of care. We aimed to develop diagnostic models to predict death ≤3 days in cancer patients.

**Methods:**

In this multicenter cohort study, we consecutively enrolled advanced cancer patients admitted to 23 inpatient hospices in 2017. Fifteen clinical signs related to impending death were documented daily from the day when the Palliative Performance Scale (PPS) declined to ≤20–14 days later. We conducted recursive partitioning analysis using the entire data set and performed cross‐validation to develop the model (prediction of 3‐day impending death‐decision tree [P3did‐DT]). Then, we summed the number of systems (nervous/cardiovascular/respiratory/musculoskeletal), where any sign was present to underpin P3did score (range = 0–4).

**Results:**

Data following PPS ≤20 were obtained from 1396 of 1896 inpatients (74%). The mean age was 73 ± 12 years, and 399 (29%) had gastrointestinal tract cancer. The P3did‐DT was based on three variables and had four terminal leaves: urine output (u/o) ≤200 ml/day and decreased response to verbal stimuli, u/o ≤200 ml/day and no decreased response to verbal stimuli, u/o >200 ml/day and Richmond Agitation‐Sedation Scale (RASS) ≤−2, and u/o >200 ml/day and RASS ≥−1. The 3‐day mortality rates were 80.3%, 53.3%, 39.9%, and 20.6%, respectively (accuracy = 68.3%). In addition, 79.6%, 62.9%, 47.2%, 32.8%, and 17.4% of patients with P3did scores of 4, 3, 2, 1, and 0, respectively, died ≤3 days.

**Conclusion:**

We successfully developed diagnostic models for death ≤3 days. These may further help clinicians predict impending death and help patients/families prepare for their final days.

## Graphical abstract

In this multicenter, prospective, observational study involving 1396 patients with advanced cancer, we successfully developed two diagnostic models for death within 3 days using physical signs. These may further help clinicians predict impending death and help patients/families prepare for the last days of life.

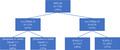

## 1 INTRODUCTION

Accurately predicting impending death is essential for terminally ill cancer patients.^1^, ^2^ It can help patients, families, and clinicians clarify goals of care, promote shared decision‐making toward the end of life (EOL), ensure goal‐concordant care, and prepare patients and families to fulfill any unfinished business and achieve a good death.^1^, ^2^, ^3^, ^4^ Such prediction becomes especially relevant and important for patients who are considered close to death based on clinical signs such as decreased activities and oral intake (e.g., Palliative Performance Scale [PPS] ≤20).^5^ Although accurate prediction of impending death among patients who start to show such “early signs” remains challenging, it would help clinicians urgently expedite EOL decision‐making.^5^ Thus, the development of validated clinical tools would be of paramount importance in this population.

Prior studies have comprehensively identified signs of impending death.^5^, ^6^, ^7^, ^8^ Hui et al. explored the diagnostic performance of multiple impending death signs and classified them into early and late impending death signs.^5^, ^8^ They also proposed a preliminary diagnostic model consisting of two signs (PPS and drooping of nasolabial folds) to predict death ≤3 days by utilizing recursive partitioning analysis.^9^ In addition, the OPCARE9 project conducted an international Delphi study and proposed several systems (e.g., decrease in consciousness, changes in respiratory status) to systematically identify patients with impending death.^7^

However, the prior studies were limited by relatively small sample sizes (up to a few hundred patients) involving single or two sites, specific settings of acute palliative care units (APCUs), where 27%–66% of patients were discharged alive, and the inclusion of patients with a relatively good performance status in whom the prediction of death ≤3 days might not always be clinically relevant.^5^, ^6^, ^8^ The natural dying process could be better observed in the setting of inpatient hospices/palliative care units (PCUs), where the majority of patients would show PPS ≤20 and subsequently die during admission. Yet, no large, multicenter cohort studies have been conducted at inpatient hospices/PCUs to develop diagnostic models of death ≤3 days when patients show PPS ≤20. In addition, while a system‐based prediction is clinically useful, how best to combine these systems has never been empirically confirmed in a multicenter study.

Our clinical questions are how clinicians can utilize the combination of individual signs or systems when death approaches at inpatient hospices/PCUs. Thus, the aim of this study was to develop diagnostic models to predict death ≤3 days in cancer patients whose PPS scores became ≤20 at inpatient hospices/PCUs. We adopted two approaches: the first was to develop a model with a decision tree and the second was to develop a system‐based score.

## 2 METHODS

### 2.1 Study setting and participants

This was part of the multicenter, prospective observational study (East‐Asian collaborative cross‐cultural Study to Elucidate the Dying process [EASED])^10^ and was the primary study conducted in Japan. We consecutively enrolled patients with advanced cancer admitted to 23 inpatient hospices/PCUs in Japan from January 1, 2017 to December 31, 2017 and followed them until their death or 6 months after their enrollment, whichever came first. All participating sites were asked to take a sample of data consecutively, up to the designed number of patients of 50, 60, 70, 80, 100, 150, and 250 according to the size of the palliative care service. This study followed the ethical standards of the Helsinki Declaration and the guidelines for medical and health research involving human subjects presented by the Japanese Ministry of Health, Labour and Welfare. Written consent was waived according to the Japanese guideline for a noninvasive observational study such as this one. The Institutional Review Boards of all participating sites approved this study.

We included patients aged 18 years or older who were diagnosed with locally advanced or metastatic cancer and admitted to inpatient hospices/PCUs in the main study. The exclusion criteria were patients who were scheduled for discharge ≤1 week or patients who declined to participate. For the current analysis, those with PPS ≤20 (i.e., bedbound, completely dependent) were included. Patients were not included if (1) they were discharged alive without developing PPS ≤20 or (2) they showed PPS ≤20 but died on the same day, as death would occur prior to the daily evaluation in the evening. PPS is a valid and reliable scale ranging from 0% (death) to 100% (completely asymptomatic) that includes the patient's function, oral intake, and cognitive status.^11^, ^12^, ^13^, ^14^ A score of ≤20% signifies that the patient is completely bedbound and has limited survival.^5^, ^15^ We decided to choose PPS ≤20 as an inception point, as the prediction of death ≤3 days in individuals with a limited performance status would be more clinically relevant than that in anyone admitted to inpatient hospices/PCUs, and PPS ≤20 could be easily detected in routine practice.^5^

### 2.2 Measurements

We selected 15 signs associated with impending death based on the previous studies, the prevalence in the literature, and ease of detection at the bedside.^5^, ^6^, ^7^, ^8^, ^9^ These included decreased level of consciousness, dysphagia of liquid; decreased response to verbal/visual stimuli, apnea periods, Cheyne‐Stokes breathing, peripheral cyanosis, pulselessness of radial artery, respiration with mandibular movement, drooping of nasolabial folds, hyperextension of the neck, inability to close eyelids, grunting of vocal cords; and decreased urine output (u/o), death rattle. The definitions of these signs were explicitly determined prior to the enrollment according to the previous study in collaboration with its principal investigator (Table S1) and extensively discussed with participating physicians to ensure interrater reliability.^5^, ^8^, ^9^ The consciousness level was recorded using the Japanese version of the Richmond Agitation‐Sedation Scale (RASS), a validated 10‐point scale ranging from −5 (unarousable) to +4 (very agitated).^16^, ^17^, ^18^ We considered an RASS score of ≤−2 to show a decreased level of consciousness.^5^

We collected data on patients’ baseline characteristics on admission to inpatient hospices/PCUs, including age, sex, marital status, tumor sites, metastases, and comorbidities.^19^ When patients’ PPS declined to ≤20, their primary responsible palliative care physicians started documenting the 15 clinical signs daily (at the end of the working hour) until death or 14 days later, whichever came first. Participating physicians from all the study institutions attended an orientation to review the study aims, design, and case report forms. In addition, the principal investigator and responsible investigators at each institution provided continuous support during the study period to ensure accurate data collection. We followed the vital status of patients until 6 months after enrollment.

### 2.3 Statistical analyses

We conducted descriptive statistics to summarize the baseline characteristics. We calculated the frequency of each sign and the median onset from death backward for all patients who died ≤14 days after the development of PPS ≤20. The median time of death after the first occurrence of each sign was estimated by the Kaplan–Meier method, conditional on observation of that particular sign. Patients who were still alive on day 14 after the development of PPS ≤20 were censored.

We encoded the diagnostic test result by grouping all the signs into “absent” or “present.” We calculated the sensitivity, specificity, positive likelihood ratio (LR), and negative LR for each sign of death ≤3 days with all observations from 1396 patients whose data following PPS ≤20 during the admission were obtained. Three days were chosen as the cutoff for impending death based on the prior study and clinical importance (e.g., shared decision‐making for EOL care).^1^, ^2^, ^5^, ^8^, ^9^ We used robust variances that are valid estimates to account for the multiple observations for each patient to obtain the point estimates and 95% confidence interval for each statistic.

To develop diagnostic models for impending death ≤3 days, we took two approaches. As the first approach, we conducted a recursive partitioning analysis (RPA) using the entire data set of the 15 clinical signs and conducted 10‐fold cross‐validation. We set the optimal tree size as one with four terminal nodes (i.e., leaves) to ensure clinical utility.^9^ To explore if the previous preliminary diagnostic model could be reproduced in our population, we also documented the proportion of patients who died ≤3 days based on drooping of nasolabial folds (presence or absence) and the number of late signs (≥2 vs. 0–1).^9^ Late signs included in the latter analysis were those used in the previous study (i.e., decreased response to verbal/visual stimuli, Cheyne‐Stokes breathing, peripheral cyanosis, pulselessness of radial artery, respiration with mandibular breathing, hyperextension of the neck, inability to close eyelids, grunting of vocal cords, death rattle, and drooping of nasolabial folds); however, nonreactive pupils and upper gastrointestinal bleed were not included in our study, as these were not routinely assessed in a daily practice of participating inpatient hospices/PCUs.^9^

As the second approach, we categorized 10 representative bedside signs into four systems based on prior studies and discussions among the researchers: nervous (decreased level of consciousness as indicated by RASS ≤−2), cardiovascular (peripheral cyanosis, pulselessness of radial artery, and decreased u/o), respiratory (apnea, Cheyne‐Stokes breathing, and respiration with mandibular movement), and musculoskeletal (inability to close eyelids, hyperextension of the neck, and drooping of nasolabial folds) systems.^5^, ^7^, ^8^, ^9^ If any sign was present within each system, a score of 1 was given to the system without a weight being assigned (i.e., each system would have a score of 0 or 1). The total score was calculated by adding the score of each system which ranges from 0 to 4 with a higher score signifying the presence of clinical signs in more systems. We also computed the sensitivity, specificity, and positive/negative LRs based on different cutoff points.

Comparison of the accuracy between the two models was considered outside our scope, as both would be of clinical use regardless of their differences in diagnostic properties. We used SAS software, version 9.4 (SAS Institute) for all statistical analyses including RPA analysis.

## 3 RESULTS

### 3.1 Patient characteristics

**FIGURE 1:**
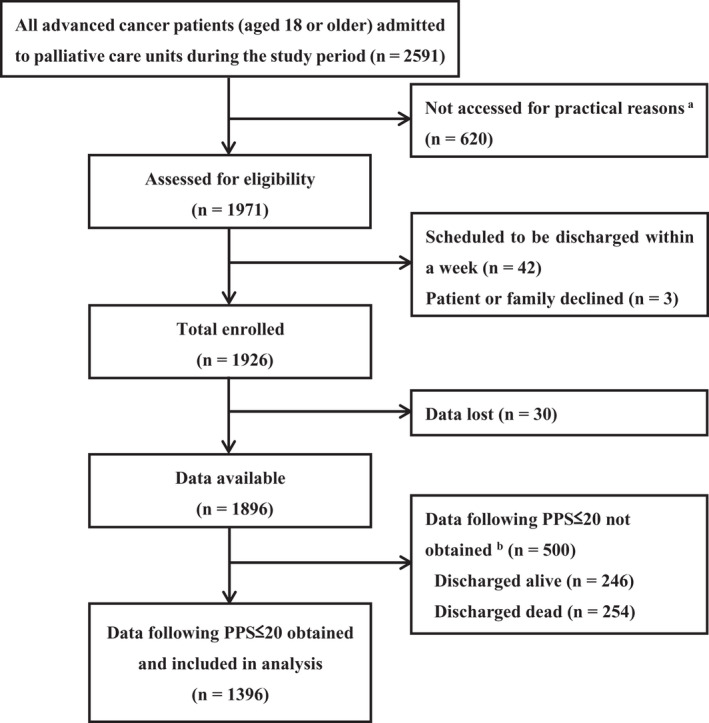
A study flow diagram. ^a^Practical reasons included unavailability of researchers on certain days of the week, outside office hours, or because of staff rotations. ^b^Data following PPS ≤20 were not obtained if patients’ PPS remained ≥30 and they were discharged alive, or if they developed PPS ≤20 and died on the same day, as death would occur prior to the daily evaluation. PPS, Palliative Performance Scale

In total, 1896 patients were included in the main study. Data following PPS ≤20 were obtained from 1396 (73.6%) and included for analysis in the current study (Figure 1). Table 1 summarizes the patients’ baseline characteristics. The mean age was 73 years, 683 (48.9%) were women, and 399 (28.6%) had gastrointestinal tract cancer. After reaching PPS ≤20, 1198 (85.8%) patients died ≤14 days, and the median length of survival was 4 days (interquartile range, 2–9).

**TABLE 1:** Baseline characteristics

Variables	*n* = 1396
Age, average (standard deviation)	72.7 (12.2)
Sex, female, *n* (%)	683 (48.9%)
Marital status, *n* (%)	
Married	833 (60.0%)
Unmarried/widowed/separated	556 (40.0%)
Cancer, *n* (%)	
Esophagus/stomach/small intestine/colorectum	399 (28.6%)
Liver/pancreas/bile duct/gallbladder	265 (19.0%)
Lung	240 (17.2%)
Kidney/ureter/bladder/prostate/testis	101 (7.2%)
Breast	93 (6.7%)
Ovary/uterus/cervix	82 (5.9%)
Head and neck	55 (3.9%)
Blood/lymph node	39 (2.8%)
Other	122 (8.7%)
Metastases, *n* (%)	
Any	1199 (85.9%)
Liver	554 (39.7%)
Lung	521 (37.4%)
Bone	372 (26.7%)
Central nervous system	199 (14.3%)
Comorbidities, *n* (%)	
Moderate–severe liver dysfunction	155 (11.1%)
Dementia	125 (9.0%)
Cerebrovascular disease	98 (7.0%)
Chronic pulmonary disorder	80 (5.7%)
Mild liver dysfunction	34 (2.4%)
Myocardial infarction	33 (2.4%)
Congestive heart failure	33 (2.4%)
Mild‐severe liver dysfunction	29 (2.1%)
Diabetes mellitus	28 (2.0%)
Peptic ulcer	24 (1.7%)
Moderate–severe renal dysfunction	24 (1.7%)
Collagen vascular disease	22 (1.6%)
Paralysis	19 (1.4%)
Peripheral vascular disease	18 (1.3%)
Acquired immunodeficiency syndrome	0
Length of survival after the first day of PPS ≤20, days, median (IQR)	5 (3–10)

Abbreviations: IQR, interquartile range; PPS, Palliative Performance Scale.

### 3.2 Frequency, onset, and diagnostic performance

Table S2 demonstrates the frequency of the 15 signs during the last week of life. Two signs (RASS ≤−2 and dysphagia of liquids) were recorded in a majority of patients in the last week of life, appearing in approximately 80% of patients 1 day prior to death. In contrast, 13 other signs were recorded in less than half of patients, even 1 day before death, except for a decreased response to visual stimuli.

Table S3 shows the median onset and diagnostic performance of the 15 signs in the prediction of death ≤3 days. The median onset of the two signs (RASS ≤−2 and dysphagia of liquids) was 3 days prior to death, and that of the other 13 signs was 1–2 days except for the death rattle. The former two signs showed high sensitivity (>70%) but low specificity for impending death ≤3 days. The latter 13 signs showed low sensitivity but high specificity (>70%). Of note, specificity and positive LR of respiration with the mandibular movement for 3‐day mortality were 98.8 (95% CI, 98.2–99.5) and 9.27 (5.45–15.78), respectively.

### 3.3 A diagnostic model: The prediction of 3‐day impending death‐decision tree

**FIGURE 2:**
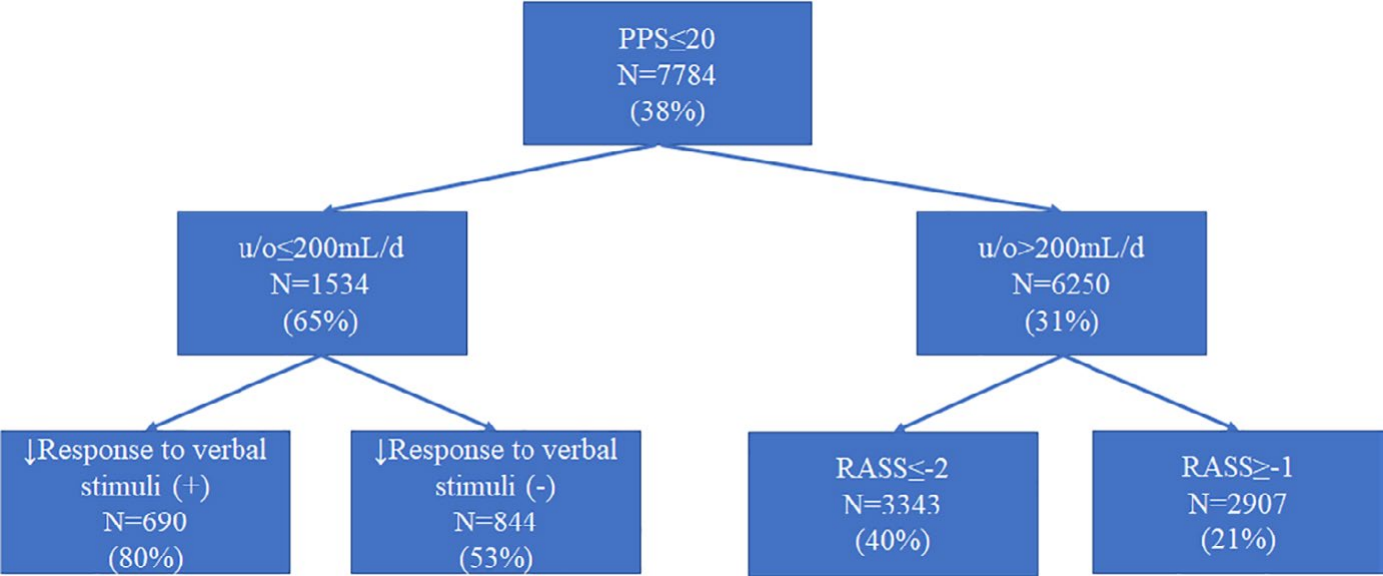
A recursive partitioning model for impending death within 3 days in patients who developed PPS ≤20 during admission at palliative care units: P3did‐DT. The main model included three variables and had two levels and four leaves. The diagnostic accuracy (1 − error rate) was 68.3%. For each node, the number of patients meeting the criteria is documented along with the 3‐day mortality rate. d, day; P3did‐DT, prediction of 3‐day impending death‐decision tree; PPS, Palliative Performance Scale; RASS, Richmond Agitation‐Sedation Scale; u/o, urine output

Figure 2 shows the final model using three variables and four terminal leaves for death ≤3 days (prediction of 3‐day impending death‐decision tree [P3did‐DT]). The 3‐day mortality rates among individuals with u/o ≤200 ml/day and decreased response to verbal stimuli, u/o ≤200 ml/day and no decreased response to verbal stimuli, u/o >200 ml/day and RASS ≤−2, and u/o >200 ml/day and RASS ≥−1 were 80.3%, 53.3%, 39.9%, and 20.6%, respectively.

Figure S1 shows the performance of the previously developed preliminary model for prediction of death ≤3 days using the current data set. Using all data available, 50.7% of patients with drooping of nasolabial folds died ≤3 days. Of patients without drooping, 52.0% of those with 2 or more late signs died ≤3 days, whereas 26.2% of those with 1 or no late signs died ≤3 days.

### 3.4 A system‐based prediction score: The P3did score

Table 2 shows the proportion of individuals who died ≤3 days with various P3did score points. In total, 79.6%, 62.9%, 47.2%, 32.8%, and 17.4% of patients with P3did score points of 4, 3, 2, 1, and 0, respectively, died ≤3 days.

**TABLE 2:** The proportion of patients who died ≤3 days based on the prediction of 3‐day impending death score (P3did score)

	P3did score (0–4)				
	4	3	2	1	0
Death ≤1 day	48.9%	32.3%	20.1%	10.8%	4.3%
Death ≤2 days	70.1%	51.4%	35.3%	22.6%	10.9%
Death ≤3 days	79.6%	62.9%	47.2%	32.8%	17.4%

Note

The P3did score is the sum of four systems: nervous, cardiovascular, respiratory, and musculoskeletal systems. If any sign is present within each system, a score of 1 is given to the system, with the total score ranging 0–4, and a higher score signifying a greater likelihood of death ≤3 days.

Table 3 demonstrates the diagnostic performance of the P3did score with various cutoff points. As a cut‐off point increases, the sensitivity for impending death decreases, while the specificity and positive LR increase. The specificity and positive LR among patients with a P3did score of four were 98.2% (95% CI, 97.4–99.1) and 6.43 (95% CI, 4.06–10.18), respectively (accuracy, 65.5%).

**TABLE 3:** Performance of P3did score in the prediction of 3‐day mortality (*n* = 1396)

P3did score	Frequency of score above each cutoff in last 3 days of life, %	Onset, median (interquartile range), days	Sensitivity, % (95% CI)	Specificity, % (95% CI)	Negative likelihood ratio (95% CI)	Positive likelihood ratio (95% CI)	Overall accuracy (%)
≥1	71.7	3 (2, 7)	87.0 (85.4–88.6)	37.6 (56.4–61.2)	0.35 (0.30–0.40)	1.39 (1.32–1.47)	56.2
≥2	39.3	2 (1, 5)	58.8 (56.4–61.2)	72.6 (69.4–75.8)	0.57 (0.53–0.61)	2.14 (1.91–2.40)	67.4
≥3	17.5	2 (1, 3)	31.5 (29.3–33.8)	91.0 (89.2–92.8)	0.75 (0.73–0.78)	3.51 (2.90–4.26)	68.6
4	5.4	1 (1, 2)	11.4 (9.9–13.0)	98.2 (97.4–99.1)	0.90 (0.89–0.92)	6.43 (4.06–10.18)	65.5

Abbreviations: CI, confidence interval; P3did, prediction of 3‐day impending death.

## 4 DISCUSSION

In this large multicenter study, we successfully developed two novel diagnostic models (P3did‐DT and P3did score) to predict death ≤3 days in patients with advanced cancer and with PPS ≤20 at inpatient hospices/PCUs. The most important finding was that 3‐day mortality can be predicted by a simple diagnostic model with three easily evaluable clinical signs (P3did‐DT): decreased u/o, decreased response to verbal stimuli, and decreased consciousness. These are supported by a previous study showing that all three variables are among the impending death signs.^5^ In practice, our findings indicated that clinicians may simply check u/o from nursing assessment, speak to the patient by calling his/her name, and assess the consciousness level by RASS, which can efficiently help predict impending death.

We also developed the P3did score, a novel system‐based tool to predict impending death. While the RPA helps identify clinical signs that could best distinguish individuals who are most likely to die ≤3 days, the final model does not fully address other impending death signs. In clinical practice, various clinical signs concurrently appear across several systems and have similarly important prognostic values. The P3did score prevents overreliance on an individual sign that may or may not clearly appear in a given imminently dying patient, like other prognostic scales.^14^, ^20^, ^21^ A system‐based categorization of clinical signs was suggested in previous studies, but its diagnostic properties have never been empirically confirmed.^7^, ^8^ The P3did score has demonstrated good diagnostic properties with high specificity over 90% using cutoff points of 3 and 4. Moreover, it has high clinical utility, as it can allow clinicians to make a comprehensive assessment. However, the diagnostic accuracy of our two models was lower than that reported in the previous study (82%).^9^ This may in part be explained by the difference in settings. Unlike the previous study that included all advanced cancer patients admitted to APCU, we included only those with PPS ≤20.^9^ Future efforts should be made to further improve the accuracy in this population.

Another important finding was that the characteristics of the early and late signs of impending death documented in the prior study are largely confirmed among those with PPS ≤20.^5^, ^8^ Hui et al. proposed that “early signs” were those observed relatively frequently and those with low specificity and included a decreased performance status (i.e., PPS ≤20), dysphagia of liquid, and decreased level of consciousness (i.e., RASS ≤−2). In our sample with PPS ≤20, these properties were shown in both dysphagia of liquid and decreased level of consciousness. On the other hand, “late signs” would reportedly emerge only in the final days prior to death in a smaller proportion of patients and showed high specificity for death ≤3 days, the properties of which were observed with the other 13 signs in our sample. Together, these findings confirmed that late signs can be used to assist clinicians in predicting impending death.

Notably, RPA did not reproduce the prior preliminary diagnostic model consisting of PPS ≤20 and drooping of nasolabial folds.^9^ The post‐hoc analysis in the current study revealed that these two variables and the number of “late signs” did not markedly categorize patients who are imminently dying. Potential interpretations are that our study differed from the previous one in terms of the setting (inpatient hospices/PCUs that provide EOL care to dying patients vs. APCUs that provide intensive symptom management to patients with severe distress), timing (cohort of patients with PPS ≤20 vs. those on admission), frequency of measurement (daily vs. twice‐a‐day assessment), and ethnicity of patients (Japanese vs. American).^5^, ^8^ The former two differences may have led to a higher prevalence of impending death and the underestimation of clinical signs in our study sample. With regards to ethnicity, nasolabial folds in Asian patients have been shown to become visibly shallower when they lie supine and still.^22^ Thus, the majority of our bedbound patients with PPS ≤20 may not have exhibited noticeable drooping of nasolabial folds. In fact, only 16.2% of the patients included in our study showed drooping of nasolabial folds during the last 3 days prior to death, whereas up to 78% showed drooping in the IPOD study, in which over 90% of patients were white or Hispanic.^5^, ^8^ As drooping of nasolabial folds is a relatively novel sign in the prediction of impending death, its validity and reliability should be further investigated among terminally ill patients with various ethnicities.

The current study had several strengths: a large sample size from over 20 institutions throughout the country, the use of explicit definitions of clinical signs based on the prior study,^5^, ^8^, ^9^ and the use of solid statistical analyses that led to highly clinically interpretable findings. However, several limitations should be noted. First, we included only cancer patients hospitalized to inpatient hospices/PCUs in Japan, in which interprofessional care is provided for the dying. Further studies should examine whether the dying process is similar in different settings (e.g., general ward, home) and in noncancer illnesses. Second, daily evaluation of the clinical signs by palliative care physicians may have limited the resolution of data, given that many impending death signs could develop a few hours before death.^6^ Moreover, data on clinical signs were not obtained from patients who died on the first day of PPS ≤20. Thus, the presence of impending death signs may have been underestimated. While the daily evaluation allowed physicians to report based on observation throughout the day, even on signs that develop intermittently, they were not asked to record how many points of assessments had occurred throughout the day. Thus, we were unable to characterize the variability in the number of assessments across days, patients, sites, and investigators. Third, the interrater reliability of the clinical signs was not formally evaluated, which may have negatively impacted the reproducibility, consistency, and validity of the models. However, participating physicians received guidance before the enrollment and continuous support during the study. Fourth, this study focused on 15 signs that were considered clinically important and relevant. The inclusion of other signs could have led to a different model. Fifth, as we included only patients who showed PPS ≤20 during admission, our findings may not be applicable to those with a relatively good condition who experience sudden deterioration and die unexpectedly. In addition, the utility of the clinical signs might be different in patients with a prolonged period of survival after the onset of PPS ≤20 than in those who develop PPS ≤20 and die shortly thereafter. Overall, we believe that our findings merit further exploration including external validation efforts with modifications to the study design to account for these limitations.

## 5 CONCLUSION

Using clinical signs, we successfully developed diagnostic models for death ≤3 days in patients with PPS ≤20. These may help clinicians predict impending death and help patients and families better prepare for the final days of life.

## CONFLICT OF INTEREST

None declared.

## ETHICS STATEMENT

This study followed the ethical standards of the Helsinki Declaration and the guidelines for medical and health research involving human subjects presented by the Japanese Ministry of Health, Labour and Welfare. Written consent was waived according to the Japanese guideline for a noninvasive observational study such as this one. The Institutional Review Boards of all participating sites approved this study.

## Supporting information

Appendix S1

## Data Availability

The data supporting the study results are available from the corresponding author upon reasonable request.
