# Supplementary material for: Diagnostic models for impending death in terminally ill cancer patients: A multicenter cohort study
Source: Cancer Med. 2021 Sep 29;10(22):7988–95. doi: 10.1002/cam4.4314 (PMC8607266; doi:10.1002/cam4.4314)
Supplement: Supplementary file 1 — Appendix S1 [file CAM4-10-7988-s001.docx]

**APPENDIX**

The participating study sites and site investigators of the EASED study in Japan were as follows: Satoshi Inoue, M.D. (Seirei Hospice, Seirei Mikatahara General Hospital), Naosuke Yokomichi, M.D., Ph.D. (Department of Palliative and Supportive Care, Seirei Mikatahara General Hospital), Kengo Imai, M.D. (Seirei Hospice, Seirei Mikatahara General Hospital), Tatsuya Morita, M.D. (Department of Palliative and Supportive Care, Seirei Mikatahara General Hospital), Masanori Mori, M.D. (Palliative Care Team, Seirei Mikatahara General Hospital), Hiroaki Tsukuura, M.D., Ph.D. (Department of Palliative Care, TUMS Urayasu Hospital), Toshihiro Yamauchi, M.D. (Seirei Hospice, Seirei Mikatahara General Hospital), Akemi Shirado Naito, M.D. (Department of Palliative Care Miyazaki Medical Association Hospital), Yu Uneno, M.D. (Department of Therapeutic Oncology, Graduate School of Medicine, Kyoto University), Akira Yoshioka, M.D., Ph.D. (Department of Oncology and Palliative Medicine, Mitsubishi Kyoto Hospital), Shuji Hiramoto, M.D. (Department of Oncology and Palliative Medicine, Mitsubishi Kyoto Hospital), Ayako Kikuchi, M.D. (Department of Oncology and Palliative Medicine, Mitsubishi Kyoto Hospital), Tetsuo Hori, M.D. (Department of Respiratory surgery, Mitsubishi Kyoto Hospital), Yosuke Matsuda, M.D. (Palliative Care Department, St. Luke's International Hospital), Hiroyuki Kohara, M.D., Ph.D. (Hiroshima Prefectural Hospital), Hiromi Fanaki, M.D. (Hiroshima Prefectural Hospital), Keiko Tanaka, M.D., Ph.D. (Department of Palliative Care Tokyo Metropolitan Cancer & Infectious Diseases Center Komagome Hospital), Kozue Suzuki, M.D. (Department of Palliative Care Tokyo Metropolitan Cancer & Infectious Diseases Center Komagome Hospital), Tina Kamei, M.D. (Department of Palliative Care, NTT Medical Center Tokyo), Yukari Azuma, M.D. (Home Care Clinic Aozora Shin-Matsudo), Koji Amano, M.D. (Department of Palliative Medicine, Osaka City General Hospital), Teruaki Uno, M.D. (Department of Palliative Medicine, Osaka City General Hospital), Jiro Miyamoto, M.D. (Department of Palliative Medicine, Osaka City General Hospital), Hirofumi Katayama, M.D. (Department of Palliative Medicine, Osaka City General Hospital), Hideyuki Kashiwagi, M.D., MBA. (Aso Iizuka Hospital / Transitional and Palliative Care), Eri Matsumoto, M.D. (Aso Iizuka Hospital / Transitional and Palliative Care), Kiyofumi Oya, M.D. (Aso Iizuka Hospital / Transitional and Palliative Care), Takeya Yamaguchi, M.D. (Japan Community Health care Organization Kyushu Hospital / Palliative Care), Tomonao Okamura, M.D., MBA. (Aso Iizuka Hospital / Transitional and Palliative Care), Hoshu Hashimoto, M.D., MBA. (Inoue Hospital / Internal Medicine), Shunsuke Kosugi, M.D. (Department of General Internal Medicine, Aso Iizuka Hospital), Nao Ikuta, M.D. (Department of Emergency Medicine, Osaka Red Cross Hospital), Yaichiro Matsumoto, M.D. (Department of Transitional and Palliative Care, Aso Iizuka Hospital), Takashi Ohmori, M.D. (Department of Transitional and Palliative Care, Aso Iizuka Hospital), Takehiro Nakai, M.D. (Immuno-Rheumatology Center, St. Luke's International Hospital), Takashi Ikee, M.D. (Department of Cardiorogy, Aso Iizuka Hospital), Yuto Unoki, M.D. (Department of General Internal Medicine, Aso Iizuka Hospital), Kazuki Kitade, M.D. (Department of Orthopedic Surgery, Saga-Ken Medical Centre Koseikan), Shu Koito, M.D. (Department of General Internal Medicine, Aso Iizuka Hospital), Nanao Ishibashi, M.D. (Environmental Health and Safety Division, Environmental Health Department, Ministry of the Environment), Masaya Ehara, M.D. (TOSHIBA), Kosuke Kuwahara, M.D. (Department of General Internal Medicine, Aso Iizuka Hospital), Shohei Ueno, M.D. (Department of Hematology / Oncology, Japan Community Healthcare Organization Kyushu Hospital), Shunsuke Nakashima, M.D. (Oshima Clinic), Yuta Ishiyama, M.D. (Department of Transitional and Palliative Care, Aso Iizuka Hospital), Akihiro Sakashita, M.D., Ph.D. (Department of Palliative Medicine, Kobe University School of Medicine), Ryo Matsunuma, M.D. (Department of Palliative Medicine, Kobe University Graduate School of Medicine), Hana Takatsu, M.D. (Division of Palliative Care, Konan Medical Center), Takashi Yamaguchi, M.D., Ph.D. (Division of Palliative Care, Konan Medical Center), Satoko Ito, M.D. (Hospice, The Japan Baptist Hospital), Toru Terabayashi, M.D. (Hospice, The Japan Baptist Hospital), Jun Nakagawa, M.D. (Hospice, The Japan Baptist Hospital), Tetsuya Yamagiwa, M.D., Ph.D. (Hospice, The Japan Baptist Hospital), Akira Inoue, M.D., Ph.D. (Department of Palliative Medicine Tohoku University School of Medicine), Takuhiro Yamaguchi, Ph.D. (Professor of Biostatistics, Tohoku University Graduate School of Medicine), Mitsunori Miyashita, R.N., Ph.D. (Department of Palliative Nursing, Health Sciences, Tohoku University Graduate School of Medicine), Saran Yoshida, Ph.D. (Graduate School of Education, Tohoku University), Yusuke Hiratsuka, M.D., Ph.D. (Department of Palliative Medicine Tohoku University School of Medicine), Keita Tagami, M.D., Ph.D. (Department of Palliative Medicine Tohoku University School of Medicine), Hiroaki Watanabe, M.D. (Department of Palliative Care, Komaki City Hospital), Takuya Odagiri, M.D. (Department of Palliative Care, Komaki City Hospital), Tetsuya Ito, M.D., Ph.D. (Department of Palliative Care, Japanese Red Cross Medical Center), Masayuki Ikenaga, M.D. (Hospice, Yodogawa Christian Hospital), Keiji Shimizu, M.D., Ph.D. (Department of Palliative Care Internal Medicine, Osaka General Hospital of West Japan Railway Company), Akira Hayakawa, M.D., Ph.D. (Hospice, Yodogawa Christian Hospital), Rena Kamura, M.D. (Hospice, Yodogawa Christian Hospital), Takeru Okoshi, M.D., Ph.D. (Okoshi Nagominomori Clinic), Isseki Maeda M.D., Ph.D. (Department of Palliative Care, Senri-Chuo Hospital), Tomohiro Nishi, M.D. (Kawasaki Municipal Ida Hospital, Kawasaki Comprehensive Care Center), Kazuhiro Kosugi, M.D. (Department of Palliative Medicine, National Cancer Center Hospital East), Yasuhiro Shibata, M.D. (Kawasaki Municipal Ida Hospital, Kawasaki Comprehensive Care Center), Takayuki Hisanaga, M.D. (Department of Palliative Medicine, Tsukuba Medical Center Hospital), Takahiro Higashibata, M.D., Ph.D. (Department of General Medicine and Primary Care, Palliative Care Team, University of Tsukuba Hospital), Ritsuko Yabuki, M.D. (Department of Palliative Medicine, Tsukuba Medical Center Hospital), Shingo Hagiwara, M.D., Ph.D. (Department of Palliative Medicine, Yuai Memorial Hospital), Miho Shimokawa, M.D. (Department of Palliative Medicine, Tsukuba Medical Center Hospital), Satoshi Miyake, M.D., Ph.D. (Professor, Department of Clinical Oncology Graduate School of Medical and Dental Sciences Tokyo Medical and Dental University (TMDU)), Junko Nozato, M.D. (Specially Appointed Assistant Professor, Department of Internal Medicine, Palliative Care, Medical Hospital, Tokyo Medical and Dental University), Hiroto Ishiki, M.D. (Department of Palliative Medicine, National Cancer Center Hospital), Tetsuji Iriyama, M.D. (Specially Appointed Assistant Professor, Department of Internal Medicine, Palliative Care, Medical Hospital, Tokyo Medical and Dental University), Keisuke Kaneishi, M.D., Ph.D. (Department of Palliative Care Unit, JCHO Tokyo Shinjuku Medical Center), Mika Baba, M.D., Ph.D. (Department of Palliative Medicine, Suita Tokushukai Hospital), Tomofumi Miura, M.D., Ph.D. (Department of Palliative Medicine, National Cancer Center Hospital East), Yoshihisa Matsumoto, M.D., Ph.D. (Department of Palliative Medicine, National Cancer Center Hospital East), Ayumi Okizaki, Ph.D. (Department of Palliative Medicine, National Cancer Center Hospital East), Yuki Sumazaki Watanabe, M.D. (Department of Palliative Medicine, National Cancer Center Hospital East), Yuko Uehara, M.D. (Department of Palliative Medicine, National Cancer Center Hospital East), Eriko Satomi, M.D. (Department of Palliative Medicine, National Cancer Center Hospital), Kaoru Nishijima, M.D. (Department of Palliative Medicine, Kobe University Graduate School of Medicine), Junichi Shimoinaba, M.D. (Department of Hospice Palliative Care, Eikoh Hospital), Ryoichi Nakahori, M.D. (Department of Palliative Care, Fukuoka Minato Home Medical Care Clinic), Takeshi Hirohashi, M.D. (Eiju General Hospital), Jun Hamano, M.D., Ph.D. (Assistant Professor, Faculty of Medicine, University of Tsukuba), Natsuki Kawashima, M.D. (Department of Palliative Medicine, Tsukuba Medical Center Hospital), Takashi Kawaguchi, Ph.D. (Tokyo University of Pharmacy and Life Sciences Department of Practical Pharmacy), Megumi Uchida, M.D., Ph.D. (Dept. of Psychiatry and Cognitive-Behavioral Medicine, Nagoya City University Graduate School of Medical Sciences), Ko Sato, M.D., Ph.D. (Hospice, Ise Municipal General Hospital), Yoichi Matsuda, M.D., Ph.D. (Department of Anesthesiology & Intensive Care Medicine / Osaka University Graduate School of Medicine), Yutaka Hatano, M.D., Ph.D. (Hospice, Gratia Hospital), Satoru Tsuneto, M.D., Ph.D. (Professor, Department of Human Health Sciences, Graduate School of Medicine, Kyoto University Department of Palliative Medicine, Kyoto University Hospital), Sayaka Maeda, M.D. (Department of Palliative Medicine, Kyoto University Hospital), Yoshiyuki Kizawa M.D., Ph.D., FJSIM, DSBPMJ. (Designated Professor and Chair, Department of Palliative Medicine, Kobe University School of Medicine), Hiroyuki Otani, M.D. (Palliative Care Team, and Palliative and Supportive Care, National Kyushu Cancer Center).

**Supplemental figure and tables**

**Supplemental Figure 1. Validation of the Investigating the Process of Dying study model: Application of drooping of nasolabial folds and the number of late signs.**

PPS≤20%

n=7786

Drooping-positive

n=1372 (50.7%)

Drooping-negative

n=6414 (35.0%)

No. of late signs ≥2

n=2181 (52.0%)

No. of late signs 0-1

n=4233 (26.2%)

Footnote. Late signs included here are those used in the previous study (Hui D, et al. Cancer 2015) except for non-reactive pupils and upper gastrointestinal bleed: decreased response to verbal stimuli, decreased response to visual stimuli, Cheyne-Stokes breathing, peripheral cyanosis, pulselessness of radial artery, respiration with mandibular breathing, hyperextension of neck, inability to close eyelids, grunting of vocal cords, death rattle, and drooping of nasolabial folds.

**Supplemental Table 1. Definition of clinical signs**

| **Physical sign** | **Description** | **Criteria for negative sign** | **Criteria for positive sign** |
| --- | --- | --- | --- |
| Decreased level of consciousness | Richmond Agitation Sedation Scale (RASS) | -1 to +4 | -2 to -5 (sedation) |
| Dysphagia of liquid | Difficulty with fluid intake | 0: Absent | 1: Present |
| Decreased response to verbal stimuli | Inability to follow verbal instructions | 0: Response present | 1: Response absent |
| Decreased response to visual stimuli | Lack of eye movements to follow things | 0: Response present | 1: Response absent |
| Apnea periods | Prolonged pauses between each breath | 0: None | 1: ≥10 seconds |
| Cheyne-Stokes breathing | Alternating periods of apnea and hyperpnea with a crescendo-decrescendo pattern | 0: Absent | 1: Present |
| Peripheral cyanosis | Bluish discoloration of extremities | 0: Absent | 1: Present |
| Pulselessness of radial artery | Inability to palpate radial pulse | 0: Palpable | 1: Non-palpable |
| Respiration with mandibular movement | Depression of jaw with inspiration | 0: Absent | 1: Present |
| Drooping of nasolabial folds | Decrease in prominence/visibility of nasolabial folds | 0: Decrease absent | 1: Decrease present |
| Hyperextension of neck | Extension of the neck backward | 0: Absent | 1: Present |
| Inability to close eyelids | Eyes are kept open with no eye blink | 0: Absent | 1: Present |
| Grunting of vocal cords | Sounds associated with vocal cord vibration mainly on expiration | 0: Absent | 1: Present |
| Decreased urine output | Measured or estimated volume of urine over a 24-hour period | 0: >200 mL | 1: ≤200 mL |
| Death rattle | Gurgling sound produced on inspiration and/or expiration related to airway secretions | 0: None | 1: Audible if very close; audible at the end of bed; audible >6 meters from door of room |

**Supplemental Table 2. Frequency of 15 clinical signs before death**

|  | **Frequency of each sign before death, n (%) (95% CI) ^a^** | | | | | | |
| --- | --- | --- | --- | --- | --- | --- | --- |
| **Physical signs** | **-7 days**  **(n=374)** | **-6 days (n=428)** | **-5 days (n=493)** | **-4 days (n=607)** | **-3 days (n=751)** | **-2 days (n=968)** | **-1 day (n=1220)** |
| Decreased level of consciousness (RASS ≤-2) | 202/374 (54.0)  (49.0, 59.1) | 234/428 (54.7)  (50.0, 59.4) | 280/493 (56.8)  (52.4, 61.2) | 374/607 (61.6)  (57.8, 65.5) | 505/751 (67.2)  (63.9, 70.6) | 698/968 (72.1)  (69.3, 74.9) | 969/1220 (79.4)  (77.2, 81.7) |
| Dysphagia of liquid | 269/374 (71.9)  (67.4 76.5) | 312/428 (72.9)  (68.7 77.1) | 366/493 (74.2)  (70.4 78.1) | 448/607 (73.8)  (70.3 77.3) | 579/751 (77.1)  (74.1 80.1) | 783/968 (80.9)  (78.4, 83.4) | 1004/1220 (82.3)  (80.2, 84.4) |
| Decreased response to verbal stimuli | 85/374 (22.7)  (18.5 27.0) | 94/428 (22.0)  (18.0 25.9) | 125/493 (25.4)  (21.5 29.2) | 167/607 (27.5)  (24.0 31.1) | 221/751 (29.4)  (26.2 32.7) | 360/968 (37.2)  (34.2 40.2) | 607/1220 (49.8)  (47.0 52.6) |
| Decreased response to visual stimuli | 128/374 (34.2)  (29.4 39.0) | 142/428 (33.2)  (28.7 37.6) | 178/493 (36.1)  (31.9 40.4) | 237/607 (39.0)  (35.2 42.9) | 330/751 (43.9)  (40.4 47.5) | 488/968 (50.4)  (47.3 53.6) | 756/1220 (62.0)  (59.2 64.7) |
| Apnea periods | 49/374 (13.1)  (9.7 16.5) | 67/428 (15.7)  (12.2 19.1) | 93/493 (18.9)  (15.4 22.3) | 113/607 (18.6)  (15.5 21.7) | 156/751 (20.8)  (17.9 23.7) | 246/968 (25.4)  (22.7 28.2) | 364/1220 (29.8)  (27.3 32.4) |
| Cheyne-Stokes breathing | 13/374 (3.5)  (1.6 5.3) | 24/428 (5.6)  (3.4 7.8) | 33/493 (6.7)  (4.5 9.3) | 45/607 (7.4)  (5.3 9.5) | 63/751 (8.4)  (6.4 10.4) | 83/968 (8.6)  (6.8 10.3) | 152/1220 (12.5)  (10.6 14.3) |
| Peripheral cyanosis | 60/374 (16.0)  (12.3 19.8) | 67/428 (15.7)  (12.2 19.1) | 77/493 (15.6)  (12.4 18.8) | 116/607 (19.1)  (16.0 22.2) | 160/751 (21.3)  (18.4 24.2) | 249/968 (25.7)  (23.0 28.5) | 478/1220 (39.2)  (36.4 41.9) |
| Pulselessness of radial artery | 11/374 (2.9)  (1.2 4.7) | 11/428 (2.6)  (1.1 4.1) | 14/493 (2.8)  (1.4 4.3) | 20/607 (3.3)  (1.9 4.7) | 40/751 (5.3)  (3.7 6.9) | 75/968 (7.8)  (6.1 9.4) | 208/1220 (17.1)  (15.0 19.3) |
| Respiration with mandibular movement | 2/374  (0.5)  (0.06 1.9) | 4/428  (0.9)  (0.3 2.4) | 13/493 (2.6)  (1.2 4.1) | 22/607 (3.6)  (2.1 5.1) | 36/751 (4.8)  (3.3 6.3) | 76/968 (7.9)  (6.2 9.6) | 214/1220 (17.5)  (15.4 19.7) |
| Drooping of nasolabial folds | 56/374 (15.0)  (11.4 18.6) | 68/428 (15.9)  (12.4 19.4) | 84/493 (17.0)  (13.7 20.4) | 113/607 (18.6)  (15.5 21.7) | 136/751 (18.1)  (15.4 20.9) | 210/968 (21.7)  (19.1 24.3) | 350/1220 (28.7)  (26.2 31.2) |
| Hyperextension of neck | 20/374 (5.4)  (3.1 7.6) | 23/428 (5.4)  (3.2 7.5) | 32/493 (6.5)  (4.3 8.7) | 42/607 (6.9)  (4.9 8.9) | 55/751 (7.3)  (5.5 9.2) | 86/968 (8.9)  (7.1 10.7) | 139/1220 (11.4)  (9.6 13.2) |
| Inability to close eyelids | 19/374 (5.1)  (2.9 7.3) | 33/428 (7.7)  (5.2 10.2) | 41/493 (8.3)  (5.9 10.8) | 59/607 (9.7)  (7.4 12.1) | 83/751 (11.1)  (8.8 13.3) | 112/968 (11.6)  (9.6 13.6) | 197/1220 (16.2)  (14.1 18.2) |
| Grunting of vocal cords | 11/374 (2.9)  (1.2 4.7) | 17/428 (4.0)  (2.1 5.8) | 21/493 (4.3)  (2.5 6.0) | 28/607 (4.6)  (2.9 6.3) | 46/751 (6.1)  (4.4 7.8) | 83/968 (8.6)  (6.8 10.3) | 142/1220 (11.6)  (9.8 13.4) |
| Urine output over last 24 h ≤200 mL | 39/374 (10.4)  (7.3 13.5) | 61/428 (14.3)  (10.9 17.6) | 82/493 (16.6)  (13.4 19.9) | 123/607 (20.3)  (17.1 23.5) | 168/751 (22.4)  (19.4 25.4) | 274/967 (28.3)  (25.5 31.2) | 562/1219 (46.1)  (43.3 48.9) |
| Death rattle | 86/374 (23.0)  (18.7 27.3) | 109/428 (25.5)  (21.3 29.6) | 118/493 (23.9)  (20.2 27.7) | 150/607 (24.7)  (21.3 28.1) | 197/751 (26.2)  (23.1 29.4) | 287/968 (29.7)  (26.8 32.5) | 397/1220 (32.5)  (29.9 35.2) |

Abbreviations: CI, confidence interval; RASS, Richmond Agitation Sedation Scale.

a: The nominator was the number of patients with a sign of interest, the denominator was the number of patients with data at the particular time-point. The number of patients in the denominator varied because of the different durations of hospitalization among patients and missing data.

**Supplemental Table 3. Diagnostic performance of 15 clinical signs for impending death within 3 days (n=1396)**

| **Physical signs** | **Frequency of signs in last 3 days of life, % ^a^** | **Onset, median (IQR), days ^b^** | **Sensitivity (%),**  **(95% CI) ^c^** | **Specificity (%)**  **(95% CI) ^c^** | **Negative LR**  **(95% CI) ^c^** | **Positive LR**  **% (95% CI) ^c^** |
| --- | --- | --- | --- | --- | --- | --- |
| Decreased level of consciousness (RASS ≤-2) | 80.4 | 3 (2, 7) | 73.9  (71.8, 76.0) | 52.3  (48.7, 55.9) | 0.50  (0.45, 0.55) | 1.55  (1.44, 1.67) |
| Dysphagia of liquid | 75.0 | 3 (2, 8) | 80.5  (78.4, 82.6) | 33.8 (30.0, 37.7) | 0.58  (0.50, 0.66) | 1.22  (1.15, 1.29) |
| Decreased response to verbal stimuli | 25.1 | 2 (1, 5) | 40.4  (38.0, 42.8) | 82.1  (79.5, 84.7) | 0.73  (0.70, 0.76) | 2.26  (1.96, 2.59) |
| Decreased response to visual stimuli | 39.8 | 2 (1, 5) | 53.6  (51.1, 56.1) | 71.6  (68.3, 74.9) | 0.65  (0.61, 0.69) | 1.89  (1.69, 2.12) |
| Apnea periods | 17.4 | 2 (1, 5) | 26.1  (23.7, 28.4) | 86.7  (84.2, 89.2) | 0.85  (0.82, 0.88) | 1.96  (1.63, 2.35) |
| Cheyne-Stokes breathing | 6.6 | 2 (1, 5) | 10.1  (8.5, 11.7) | 95.4  (93.9, 96.9) | 0.94  (0.92, 0.96) | 2.19  (1.60, 3.01) |
| Peripheral cyanosis | 22.3 | 2 (1, 4) | 30.2  (27.8, 32.5) | 88.1  (85.8, 90.4) | 0.79  (0.77, 0.82) | 2.54  (2.12, 3.05) |
| Pulselessness of radial artery | 6.8 | 1 (1, 2) | 11.0  (9.5, 12.5) | 97.7  (96.5, 98.8) | 0.91  (0.90, 0.93) | 4.67  (2.88, 7.59) |
| Respiration with mandibular movement | 6.1 | 1 (1, 2) | 11.1  (9.6, 12.6) | 98.8  (98.2, 99.5) | 0.90  (0.89, 0.92) | 9.27  (5.45, 15.78) |
| Drooping of nasolabial folds | 16.2 | 2 (1, 5) | 23.7  (21.4, 25.9) | 86.1  (83.3, 88.8) | 0.89  (0.86, 0.92) | 1.70  (1.41, 2.05) |
| Hyperextension of neck | 6.6 | 2 (1, 5) | 9.5  (7.9, 11.1) | 95.7  (94.2, 97.2) | 0.95  (0.93, 0.96) | 2.23  (1.60, 3.12) |
| Inability to close eyelids | 9.0 | 2 (1, 4) | 13.3  (11.5, 15.1) | 94.5  (92.8, 96.1) | 0.92  (0.90, 0.94) | 2.40  (1.80, 3.22) |
| Grunting of vocal cords | 4.5 | 2 (1, 3) | 9.2  (7.8, 10.6) | 97.7  (96.8, 98.5) | 0.93  (0.92, 0.94) | 3.96  (2.82, 5.55) |
| Urine output over last 24 h ≤200 mL | 23.9 | 2 (1, 4) | 34.2  (31.9, 36.5) | 89.1  (87.0, 91.1) | 0.74  (0.71, 0.77) | 3.13  (2.62, 3.73) |
| Death rattle | 24.4 | 3 (2, 8) | 30.0  (27.5, 32.5) | 76.5  (73.2, 79.8) | 0.92  (0.87, 0.96) | 1.28  (1.11, 1.47) |

Abbreviations: CI, confidence interval; IQR, interquartile range; LR, likelihood ratio; RASS, Richmond Agitation Sedation Scale.

a: Any occurrence of the sign of interest within the last 3 days of life among patients who became PPS≤20 and died in the palliative care units.

b: Onset from death backwards.

c: We computed the sensitivity, specificity, positive likelihood ratio, and negative likelihood ratio for each sign of death within 3 days with all observations from 1396 patients who developed PPS≤20 during the admission at palliative care units. We constructed a 2 x 2 table with 1 observation per patient based on the presence or absence of a particular sign during a randomly sampled day and on whether that patient died within the next 3 days after that day (i.e., gold standard); we then calculated the sensitivity, specificity, positive likelihood ratio, and negative likelihood ratio. We used robust variances to account for the multiple observations for each patient to obtain the point estimates and 95% confidence interval for each statistic.
